# Identification of m6A‐related lncRNAs as prognostic signature within colon tumor immune microenvironment

**DOI:** 10.1002/cnr2.1828

**Published:** 2023-05-13

**Authors:** Lichao Cao, Shenrui Zhang, Ying Ba, Hezi Zhang

**Affiliations:** ^1^ Provincial Key Laboratory of Biotechnology of Shaanxi Province Northwest University Xi'an China; ^2^ Key Laboratory of Resource Biology and Biotechnology in Western China, Ministry of Education, School of Life Sciences Northwest University Xi'an China; ^3^ Research and Development Department Shenzhen Nucleus Gene Technology Co., Ltd. Shenzhen China

**Keywords:** colon cancer, lncRNAs, N6‐methylandenosine, prognostic model, tumor immune microenvironment

## Abstract

**Background:**

The current study probed prognosis‐related potential for m6A‐related lncRNAs signatures within colon tumor immune microenvironment (TIM).

**Methods:**

After downloading transcriptomic datasets for colon cancer (CC) patients from The Cancer Genome Atlas (TCGA), they were divided, in a 1:1 ratio, within training or test datasets. m6A‐related lncRNAs were then scrutinized across such dataset using Pearson correlation assessment before generating a m6A‐related lncRNAs prognosis‐related model using the training dataset. The latter was then validated with the test and the whole dataset. In addition, we compared the differences of TIM and the estimated IC50 of drug response between the high‐ and low‐risk groups.

**Results:**

Overall survival (OS) resulted as linked with 11 m6A‐related lncRNAs, while within the developed prognosis‐related model, areas‐under‐curves were as follows: within training dataset, values at 3‐, 4‐, and 5‐years were 0.777, 0.819, and 0.805, accordingly, and for test one, they were 0.697, 0.682, and 0.706, respectively. Finally, the values for the whole dataset were 0.675 (3‐year), 0.682 (4‐years), and 0.679 (5‐years), accordingly. Moreover, CC cases categorized within low‐risk cohort demonstrated enhanced OS (*p* < .0001), lower metastasis (*p* = 2e‐06) and lower T stage (*p* = .0067), more instability for microsatellite status (*p* = .012), and downregulation for *PD‐L1, PD‐1, CTLA‐4, LAG3*, and *HAVCR2* (*p* < .05). In addition, risk scorings were significantly linked to the degree of infiltrative intensity for CD8 and CD4 (memory resting) T‐cells, T‐regulatory (Tregs), and Mast cells triggering (*p* < .05). Patients with low infiltrative propensity for CD4 T‐cells also had better OS (*p* = .016). Moreover, six representative drugs were found to be sensitive for treating CC patients.

**Conclusion:**

A robust m6A‐related prognostic model with great performances was developed before exploring the TIM characteristics and its potential therapeutic drugs, which might improve the prognosis and therapeutic efficacy.

## 1 INTRODUCTION

In 2022, the new cases and mortality of colorectal cancer were both ranked in the top three among all cancers.^1^ So far, great progress has been made in terms of its diagnosis and treatment,^2^, ^3^ with patients who have been diagnosed at an early stage now having significantly improved prognosis.^4^ However, more efficient biomarkers still need to be identified, especially for predicting prognosis which remains poor in patients with tumor metastasizes.^5^, ^6^ In this context, a previous study noted that the tumor immune microenvironment (TIM) was essential for predicting prognosis as well as for evaluating therapeutic efficacy.^7^ For instance, immune checkpoint inhibitors, such as cytotoxic T lymphocyte antigen 4 (*CTLA4*) together with programmed cell death 1 (*PD‐1*) inhibitors, have been identified and used as antitumor agents for immunotherapy. Similarly, lymphocyte activation gene‐3 (*LAG3*) has been considered a target of immunotherapy as its up‐regulation can exhaust immune cell proliferation and cytokine production.^8^ Finally, *HAVCR2* can encode T‐cell immunoglobulin and mucin domain 3 (*Tim‐3*), a checkpoint receptor that plays vital roles within tumor microenvironment.^9^

N6‐methyladenosine (m6A) modifications are important for a number of biological functions, including mRNA handling, together with exporting, translating as well as biogenesis for long‐noncoding RNA (lncRNA) and microRNA that contribute to human carcinogenesis.^10^ Indeed, mutations or aberrant expression of lncRNAs (ncRNAs that are >200 nt long) may exhibit oncogenic functions.^11^ The m6A regulators can be divided into three types: writers, erasers and readers. Among them, “writer” is a methyltransferase complex that catalyzes m6A, “eraser” is a demethylase that removes m6A, and “reader” reads the protein to recognize m6A, and combines RNA to play roles in tumorigenesis and tumor progression.^12^

Using the Cancer Genome Atlas (TCGA) database, the current study applied bioinformatics as well as statistical analyses to identify and validate the suitability for m6A‐related lncRNAs to predict prognoses. A prognostic model for colon cancer (CC) was also established before assessing how it could be linked to patients' clinicopathological features. In addition, the TIM was characterized in terms of the type of tumor‐infiltrating cells, the tumor mutational burden (TMB) together with *CLTA4*/*LAG3/ PD‐L1/HAVCR2/ PD‐1* expression profiles. Finally, high‐risk and low‐risk groups were comparatively analyzed for determining how they differed in terms of potential drug resistance. These findings may help to identify novel therapeutic targets that could be effectively exploited against CC.

## 2 MATERIALS AND METHODS

### 2.1 Data acquiring and preparation

The GDC TCGA Colon Cancer (TCGA‐COAD) cohort on transcriptome and somatic mutation profiles were acquired through UCSC Xena platform (https://xenabrowser.net/datapages/), along with their corresponding clinical datasets. 35 acknowledged m6A regulator genes were selected from literatures,^12^, ^13^, ^14^ including regulators of readers [*FMR1, PRRC2A, ELAVL1, NXF1, LRPPRC, XRN1, IGF2BP1, SRSF10, YTHDC2, HNRNPA2B1, SRSF3, RBMX, SETD2, HNRNPC, CBLL1, YTHDF3, CPSF6, YTHDF2, NUDT21, YTHDF1, ZCCHC4, TRMT112, YTHDC1*], writers [*METTL3, WTAP, ZC3H13, KIAA1429, RBM15B, RBM15, PCIF1, METTL16, METTL14*] and erasers [*ALKBH3, ALKBH5 and FTO*]. Expression matrixes for m6A‐related genes were then extracted through transcriptome profiles, having 15 088 lncRNAs subsequently identified through a lncRNA annotation file (gencode.v36.long_noncoding_RNAs.gtf) of the GENCODE website.

### 2.2 Determining m6A‐related lncRNAs

The *limma* package in R compared normal group and tumor group and retrieved differentially expressed lncRNAs (DELs),^15^ having adjusted *p‐value* < 05 together with |log2 fold change (FC)| > 0.585, selected as thresholds for this purpose. In addition, differentially expressed m6A‐related genes were obtained using unpaired *t* tests (*p‐value* < .05). Finally, to determine how the DELs were correlated to the m6A‐related genes, Pearson correlation assessments were conducted, whereby in this case, m6A‐related lncRNAs were defined at a *p‐value* of < .001 and a |*Pearson correlation*| of > 0.5.

### 2.3 Generation and validating for m6A‐related lncRNAs prognosis‐related model in CC

Expression data of 432 CC patients in TCGA‐COAD cohort, along with their corresponding clinical information, were obtained and defined as the whole dataset. The latter was then randomly divided in a 1:1 ratio and assigned to either a training or a test dataset, with detailed information on each provided in Table 1. Using the training dataset, a prognostic risk model was then generated before subsequently evaluating it using the test data. Univariate Cox proportional hazards regression assessment was also conducted to identify m6A‐related lncRNAs being closely related to prognoses using the R package *survival* and *survminer* (*p* < .05), with overfitting avoided through least absolute shrinkage and selection operator (LASSO) evaluation using R package *glmnet*. Furthermore, independent prognostic factors and their coefficients were denoted through multivariate Cox regression assessment before calculating individual case risk scoring, using:

Risk scoring=∑Coxcoefficient of lnRNAχi×Scaled expression value of lncRNAχi.

**TABLE 1:** Detailed information of the training dataset, test dataset, and the whole dataset.

	The whole dataset			Training dataset			Test dataset		
	Alive	Death	Overall	Alive	Death	Overall	Alive	Death	Overall
	(*N* = 338)	(*N* = 94)	(*N* = 432)	(*N* = 174)	(*N* = 42)	(*N* = 216)	(*N* = 164)	(*N* = 52)	(*N* = 216)
Gender									
Female	158 (46.7%)	42 (44.7%)	200 (46.3%)	80 (46.0%)	15 (35.7%)	95 (44.0%)	78 (47.6%)	27 (51.9%)	105 (48.6%)
Male	180 (53.3%)	52 (55.3%)	232 (53.7%)	94 (54.0%)	27 (64.3%)	121 (56.0%)	86 (52.4%)	25 (48.1%)	111 (51.4%)
Age									
<=60	112 (33.1%)	24 (25.5%)	136 (31.5%)	46 (26.4%)	8 (19.0%)	54 (25.0%)	66 (40.2%)	16 (30.8%)	82 (38.0%)
>60	226 (66.9%)	70 (74.5%)	296 (68.5%)	128 (73.6%)	34 (81.0%)	162 (75.0%)	98 (59.8%)	36 (69.2%)	134 (62.0%)
Microsatellite status									
Missing	8 (2.4%)	0 (0%)	8 (1.9%)	4 (2.3%)	0 (0%)	4 (1.9%)	3 (1.8%)	1 (1.9%)	4 (1.9%)
Indeterminate	3 (0.9%)	0 (0%)	3 (0.7%)	1 (0.6%)	0 (0%)	1 (0.5%)	0 (0%)	1 (1.9%)	1 (0.5%)
MSI‐H	56 (16.6%)	16 (17.0%)	72 (16.7%)	34 (19.5%)	8 (19.0%)	42 (19.4%)	31 (18.9%)	11 (21.2%)	42 (19.4%)
MSI‐L	53 (15.7%)	19 (20.2%)	72 (16.7%)	26 (14.9%)	6 (14.3%)	32 (14.8%)	24 (14.6%)	8 (15.4%)	32 (14.8%)
MSS	218 (64.5%)	59 (62.8%)	277 (64.1%)	109 (62.6%)	28 (66.7%)	137 (63.4%)	106 (64.6%)	31 (59.6%)	137 (63.4%)
Pathologic stage									
I	69 (20.4%)	4 (4.3%)	73 (16.9%)	30 (17.2%)	2 (4.8%)	32 (14.8%)	39 (23.8%)	2 (3.8%)	41 (19.0%)
II	140 (41.4%)	26 (27.7%)	166 (38.4%)	73 (42.0%)	13 (31.0%)	86 (39.8%)	67 (40.9%)	13 (25.0%)	80 (37.0%)
III	93 (27.5%)	29 (30.9%)	122 (28.2%)	54 (31.0%)	12 (28.6%)	66 (30.6%)	39 (23.8%)	17 (32.7%)	56 (25.9%)
IV	30 (8.9%)	30 (31.9%)	60 (13.9%)	13 (7.5%)	12 (28.6%)	25 (11.6%)	17 (10.4%)	18 (34.6%)	35 (16.2%)
Missing	6 (1.8%)	5 (5.3%)	11 (2.5%)	4 (2.3%)	3 (7.1%)	7 (3.2%)	2 (1.2%)	2 (3.8%)	4 (1.9%)
AJCC‐T									
T1	9 (2.7%)	2 (2.1%)	11 (2.5%)	5 (2.9%)	1 (2.4%)	6 (2.8%)	4 (2.4%)	1 (1.9%)	5 (2.3%)
T2	71 (21.0%)	4 (4.3%)	75 (17.4%)	31 (17.8%)	2 (4.8%)	33 (15.3%)	40 (24.4%)	2 (3.8%)	42 (19.4%)
T3	229 (67.8%)	67 (71.3%)	296 (68.5%)	124 (71.3%)	30 (71.4%)	154 (71.3%)	105 (64.0%)	37 (71.2%)	142 (65.7%)
T4	28 (8.3%)	21 (22.3%)	49 (11.3%)	14 (8.0%)	9 (21.4%)	23 (10.6%)	14 (8.5%)	12 (23.1%)	26 (12.0%)
Missing	1 (0.3%)	0 (0%)	1 (0.2%)	0 (0%)	0 (0%)	0 (0%)	1 (0.6%)	0 (0%)	1 (0.5%)
AJCC‐N									
N0	219 (64.8%)	35 (37.2%)	254 (58.8%)	108 (62.1%)	16 (38.1%)	124 (57.4%)	111 (67.7%)	19 (36.5%)	130 (60.2%)
N1	76 (22.5%)	24 (25.5%)	100 (23.1%)	42 (24.1%)	7 (16.7%)	49 (22.7%)	34 (20.7%)	17 (32.7%)	51 (23.6%)
N2	43 (12.7%)	35 (37.2%)	78 (18.1%)	24 (13.8%)	19 (45.2%)	43 (19.9%)	19 (11.6%)	16 (30.8%)	35 (16.2%)
AJCC‐M									
M0	269 (79.6%)	49 (52.1%)	318 (73.6%)	140 (80.5%)	22 (52.4%)	162 (75.0%)	129 (78.7%)	27 (51.9%)	156 (72.2%)
M1	30 (8.9%)	30 (31.9%)	60 (13.9%)	13 (7.5%)	12 (28.6%)	25 (11.6%)	17 (10.4%)	18 (34.6%)	35 (16.2%)
MX	35 (10.4%)	12 (12.8%)	47 (10.9%)	19 (10.9%)	7 (16.7%)	26 (12.0%)	16 (9.8%)	5 (9.6%)	21 (9.7%)
Missing	4 (1.2%)	3 (3.2%)	7 (1.6%)	2 (1.1%)	1 (2.4%)	3 (1.4%)	2 (1.2%)	2 (3.8%)	4 (1.9%)

Abbreviation: AJCC, American Joint Committee on Cancer.

S*urvivalROC* in R was used for plotting receiver‐operating‐characteristic (ROC) curves together with quantifying area‐under‐curve (AUC) for evaluating risk scoring model's sensitivity as well as specificity. In this case, the ROC curve for which true positive and false positive showed most significant differences was selected, with the curve's turning point selected as the optimal cutoff value based on which cases were categorized into a low‐risk or a high‐risk group. Finally, by using the *survminer* package in R, overall survival (OS) endpoints across groups were comparatively analyzed based on Kaplan–Meier curves. The suitability of the prognostic model (i.e., in predicting prognosis) was also validated by plotting ROC and Kaplan–Meier curves using the test as well as the whole dataset, respectively.

Clinical variables (e.g., age, gender, advanced pathological stage, and microsatellite status) were used along with the risk score to construct a prognostic nomogram, with the latter's suitability assessed based on calibration curves as well as the concordance index (C‐index). Eventually, associations across risk scoring model and case clinical features, especially the TNM together with late‐stage pathological status, was studied depending upon *Wilcoxon* test. Otherwise, the expression of the identified prognostic m6A‐related lncRNAs between MSI‐H group and MSI‐L/MSS group was compared using unpaired *t* tests, and the lncRNAs with *p* < .05 were considered to be significantly differential expressed.

### 2.4 Comparison of immune cell infiltrative propensity across groups through whole dataset

Risk scores, calculated from the constructed risk model, were used to divide the whole dataset into low‐ or high‐risk groups. Normalized gene expression matrices were then used, along with CIBERSORT algorithm, for estimating degrees of presence across all 22 types of immune cells.^16^ Unpaired *t* tests further allowed the two groups of patients to be compared in terms of their immune environment before plotting Kaplan–Meier curves to determine how each significantly different immune cell type (*p‐value* < 0.05) was related to OS.

### 2.5 Exploring immunotherapy‐related characteristics

By using the *maftools* package in R on the whole dataset, the mutation profiles of the two groups could be visualized,^17^ with differences in terms of TMB compared using unpaired *t* test. Moreover, the *Wilcoxon* test allowed transcriptomic expression profiles for immune checkpoints together with linked ligands across high‐ together with low‐risk groups for comparative analyses.

### 2.6 Prediction and comparison of drug sensitivity within two CC groups

IC50 for cancer drug response related to CC treatment was predicted in each sample using *oncoPredict* R package,^18^ before selecting those drugs that had an average IC50 of less than 5. Subsequently, differences within response to these drugs within high‐risk together with low‐risk groups were comparatively analyzed using constructed prognostic risk score model.

## 3 RESULTS

### 3.1 Determining of m6A‐related DELs

**FIGURE 1:**
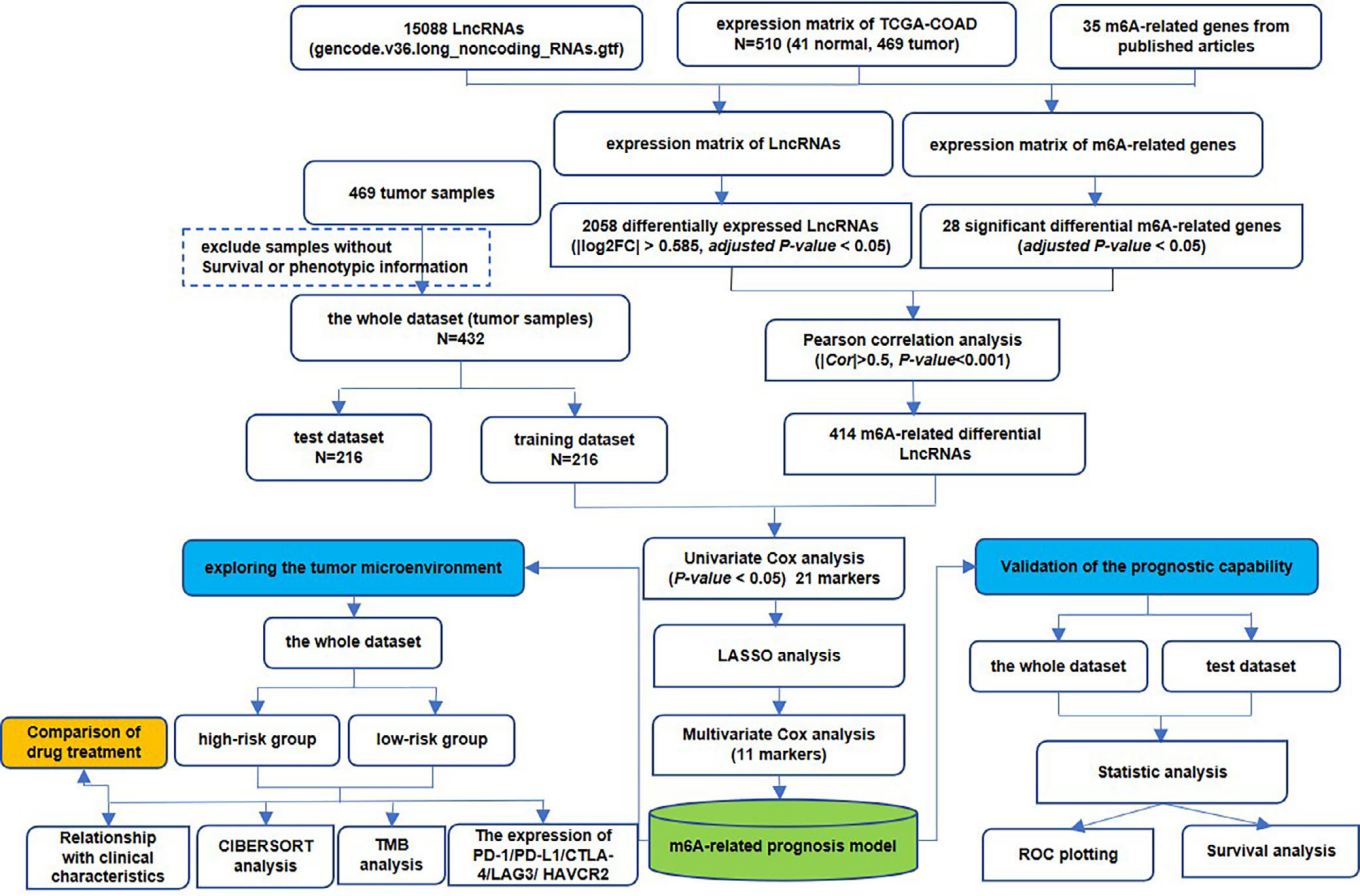
The whole flow chart of data analysis.

**FIGURE 2:**
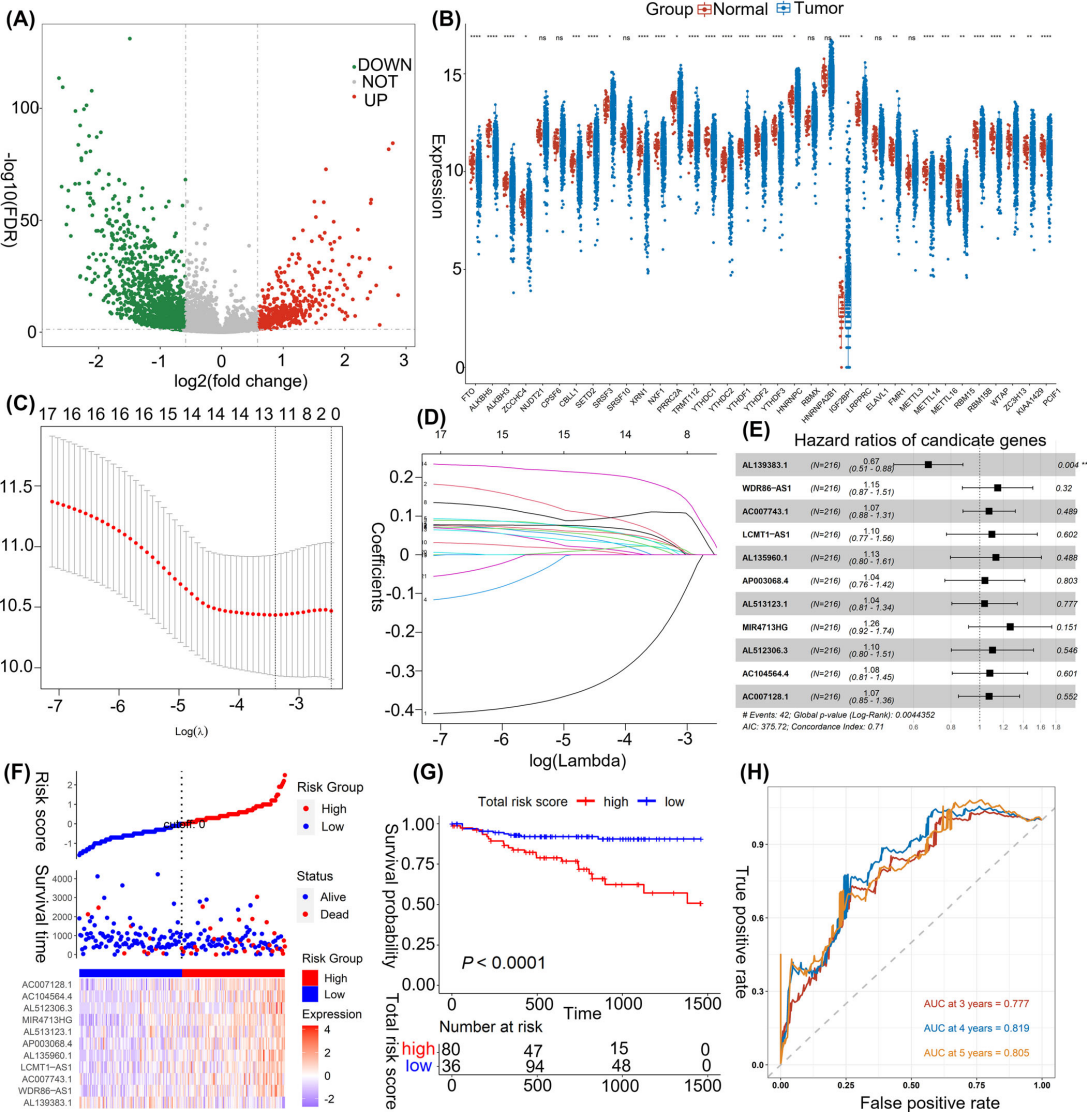
Identification of m6A‐related lncRNAs as prognostic signatures. (A) Volcano plot on all DELs between tumors and normal samples. The green dots represent downregulated lncRNAs, while the red dots represent upregulated lncRNAs. (B) Statistical differences between normal and tumor groups were compared through the unpaired t test. The *p* values are labeled above each boxplot with asterisks. (*p < .05, **p < .01, ***p < .001, ****p < .0001). (C) The optimal values of the penalty parameter λ were determined by cross‐validation. (D) LASSO coefficient profiles of the 11 lncRNAs were related with prognosis. (E) Forest plot showing the results of the multivariate Cox analysis. (F) The distribution of the high‐ and low risk score groups and its relationship with OS, and the expression pattern of 11 prognostic signatures in high‐ and low risk score groups. (G) Kaplan–Meier curve revealed that OS in the low‐risk score group was significantly higher than that in the high‐risk score group. (H) Time‐dependent ROC curve analysis of the m6A‐related risk score model.

Figure 1 highlights the whole analysis workflow. Comparisons between tumor and normal groups revealed a total of 2058 DELs (618 upregulated and 1440 downregulated) and 28 m6A‐related genes were screened (Figure 2A,B; Table S1). After Pearson correlation analysis, 414 m6A‐related DELs (114 upregulated and 300 downregulated) were obtained, with detailed information provided in Table S2.

### 3.2 Construction and prognostic value of m6A‐related risk scoring model

Prognosis‐related potential for m6A‐related DELs were identified through univariate Cox regression assessments, with 21 DELs being highly linked to OS (Table 2). Of these, LASSO modeling was used to select 11 DELs with maximum prognostic value (Figure 2C), with the coefficient of each being shown in Figure 2D. Using the training dataset, the m6A‐related prognostic model was then defined depending upon multivariate Cox regression assessment prior to forest plotting to show the hazard ratios (HR) of each prognostic indicator (Figure 2E). Eventually, this formula determined individual case risk scoring:

$$\text{Risk scoring}=\mathrm{AL}139383.1\times \left(-0.245651034\right)+\mathrm{WDR}86-\text{AS1}\times 0.069930096+\mathrm{AC}007743.1\times 0.039722371+\text{LCMT}1-\text{AS1}\times 0.040869437+\mathrm{AL}135960.1\times 0.109321350+\mathrm{AP}003068.4\times 0.022537350+\mathrm{AC}004832.5\times 0.006719394+\mathrm{AL}513123.1\times 0.022984398+\mathrm{MIR}471\text{3HG}\times 0.190161843+\mathrm{AL}512306.3\times 0.056446755+\mathrm{AC}104564.4\times 0.045905368+\mathrm{AC}007128.1\times 0.025591155.$$

**TABLE 2:** The m6a‐related lncRNAs with significant prognostic value in colon cancer identified by the univariate Cox regression analysis.

lncRNAs	HR	HR.95L	HR.95H	*p‐value*
*AL139383.1*	0.776358919	0.603375211	0.998935918	.049040206
*WDR86‐AS1*	1.311460835	1.035101084	1.661605372	.024723321
*AC007743.1*	1.203944199	1.024025997	1.415473474	.024611087
*AC003973.2*	1.235396627	1.020323142	1.495805361	.030301519
*LCMT1‐AS1*	1.369179217	1.032789671	1.81513408	.028944288
*PCOLCE‐AS1*	1.359749341	1.034795024	1.786748318	.027423595
*AL161729.4*	1.266691642	1.027345318	1.561799802	.026936283
*AL135960.1*	1.396052679	1.105332061	1.763237629	.005101367
*AC069222.1*	1.255966108	1.025676098	1.537962002	.027434626
*AC067930.4*	1.213078541	1.011564377	1.454736425	.037158712
*AP003068.4*	1.337868101	1.056639186	1.693947262	.015625778
*AC004832.5*	1.256910825	1.001974715	1.576711268	.048039258
*AL513123.1*	1.257273972	1.02762951	1.538237102	.026093617
*MIR4713HG*	1.453532341	1.126607634	1.875325716	.004014672
*AL512306.3*	1.347963763	1.056885358	1.719208515	.016140836
*AC027601.2*	1.410856523	1.043811963	1.906968113	.025163792
*AC104564.4*	1.350600002	1.048100401	1.740406133	.020174276
*AC007128.1*	1.266275002	1.020988337	1.570490401	.031630867
*AC020891.2*	1.250464906	1.009862431	1.548391575	.040366943
*FAM83C‐AS1*	1.282271832	1.00069703	1.643075778	.049359615
*FMR1‐AS1*	1.310130933	1.018940594	1.684536933	.035181015

Abbreviation: HR, hazard ratio.

After assigning patients to either low‐risk or high‐risk cohort, the former was recognized to have better OS as shown in Figure 2F,G. In particular, heatmapping (Figure 2F) indicated *AL139383.1* expression to be upregulated in comparison to low‐risk patients, with opposing expression levels for the other remaining 11 DELs. Time‐dependent ROC analysis showed that for the prognostic model, AUC in OS stood at 0.777, 0.819, and 0.805 for 3‐, 4‐, and 5‐years (Figure 2H), accordingly.

### 3.3 Validation of the constructed m6A‐related prognosis‐related model

**FIGURE 3:**
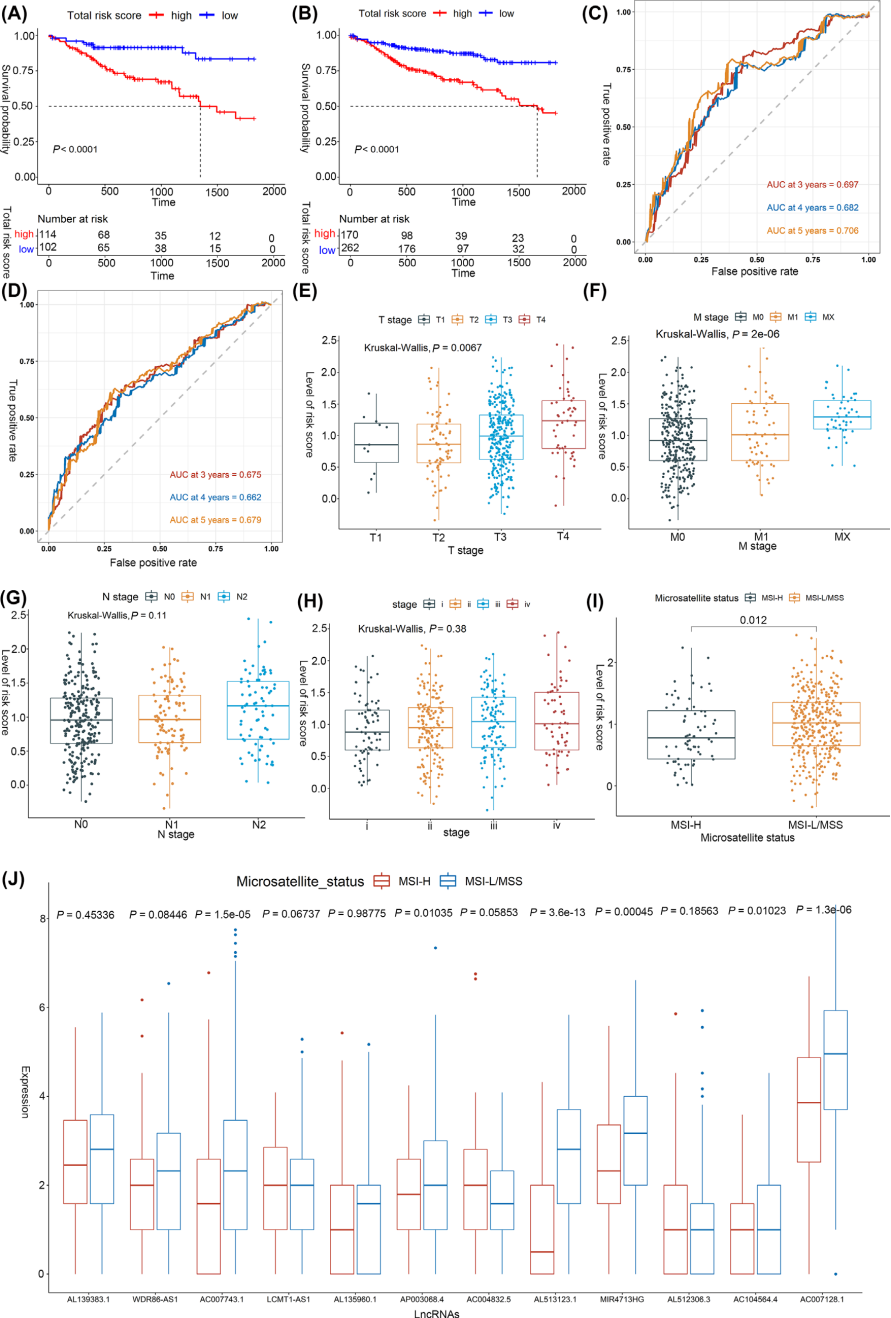
Validation of the constructed m6A‐related risk score prognostic model using test dataset (A, C) and the whole dataset (B, D). Exploring the relationship between the risk score of the colon cancer patients and the clinical pathological characteristics, including (E) T stages, (F) M stages, (G) N stages, (H) advanced pathological stages, and (I) microsatellite status, based on the whole dataset. (J) The expression of the identified prognostic m6A‐related lncRNAs between MSI‐H group and MSI‐L/MSS group.

In validating prognosis‐related potential for this novel‐established risk scoring model, individual case score within test dataset and the whole one using the identical formula. As shown by the results obtained using the training dataset, high‐risk cases exhibited poorer OS compared with the low‐risk ones (*p* < .0001) (Figure 3A,B). AUCs of the prognosis‐related model for OS were calculated (test dataset: 0.697, 0.682, and 0.706 at 3, 4, and 5‐years, respectively; the whole dataset: 0.675, 0.662, and 0.679 at 3‐, 4‐, and 5‐years) (Figure 3C,D). Based on the whole dataset, Wilcoxon test revealed increased risk scores linked to high T staging (*p* = .0067) and metastases (*p* = 2e‐06), but not highly linked to N stage (*p* = .11) or late‐stage pathological status (*p* = .38) (Figure 3E–H). However, the higher the risk score, the more stable were the microsatellite status in CC patients (*p* = .012) (Figure 3I). In addition, *AC007743.1, AP003068.4, AL513123.1, MIR4713HG, AC104564.4* and *AC007128.1* were found down‐regulated in MSI‐H group (*p* < .05), while other lncRNAs were not significantly expressed (*p* > .05) (Figure 3J).

**FIGURE 4:**
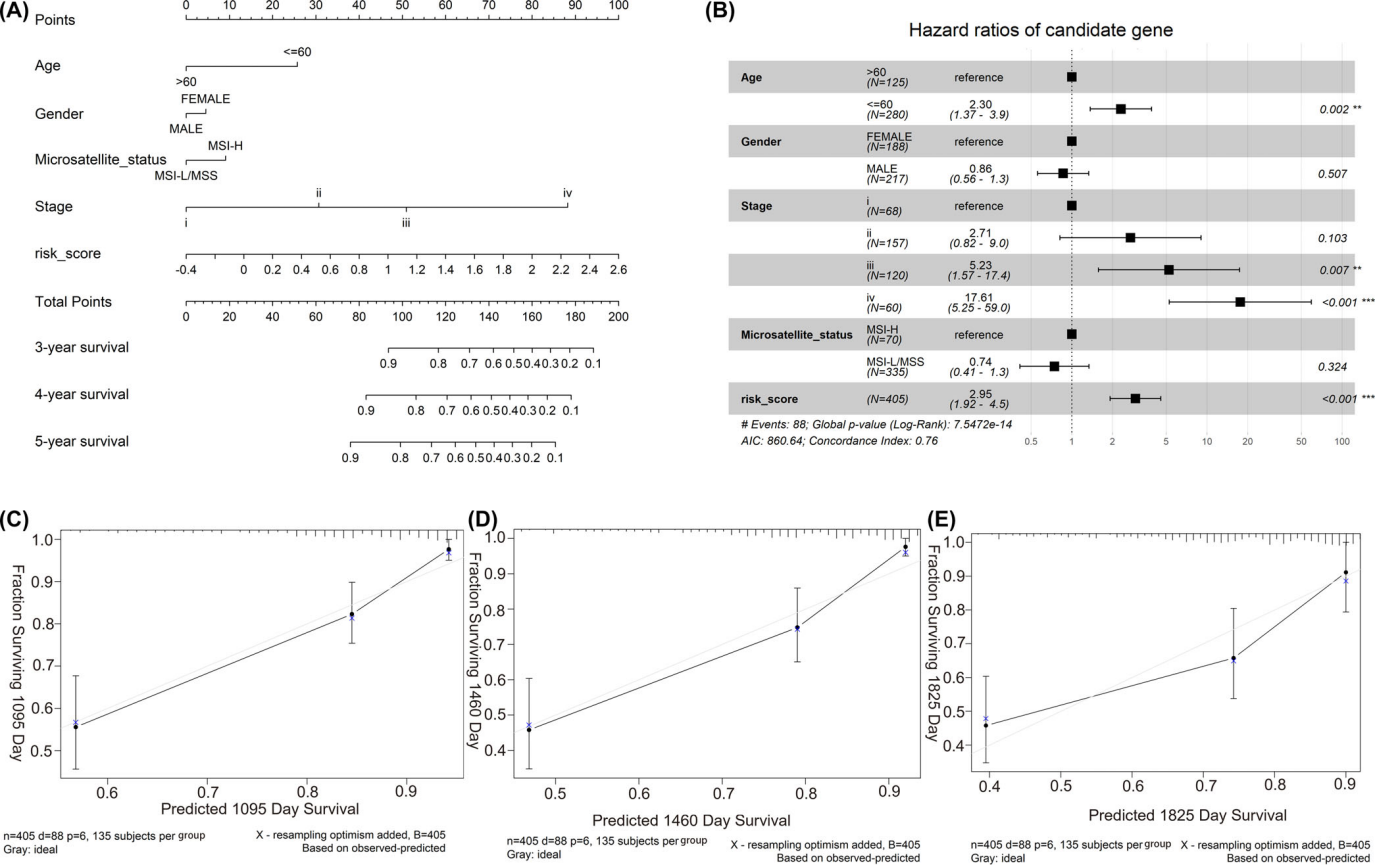
Construction and validation of a nomogram. (A) Nomogram to predict the probability of OS in 3, 4, and 5 years for colon cancer patients. (B) Forest plots showing the associations between patient's characteristics and OS. (C–E) Calibration plot of the nomogram to predict the probability of OS at 3, 4, and 5 years. ** refers to the *p* value less than 0.01, *** refers to the *p* value less than 0.001.﻿﻿﻿﻿

To further improve the prognostic model's performance, a nomogram comprising risk scoring and clinical risk characteristics was established for quantitatively predicting 3‐, 4‐, and 5‐years OS incidences based on the entire dataset. Within this nomogram, risk scoring showed predominantly predictive ability than clinical factors (Figure 4A). The forest plot showed that OS was significantly linked to risk score, tumor staging (III and IV) together with age (>60) (*p* < .05, Figure 4B). Calibration curves highlighted prediction curves to be similar to optimal curves (Figure 4C–E). Moreover, the nomogram (C‐index: 0.76, Figure 4B) had higher predictive accuracy in comparison to risk scoring model (C‐index: 0.71, Figure 2E).

### 3.4 Exploring the TIM in patients for entire dataset depending upon constructed prognostic model

**FIGURE 5:**
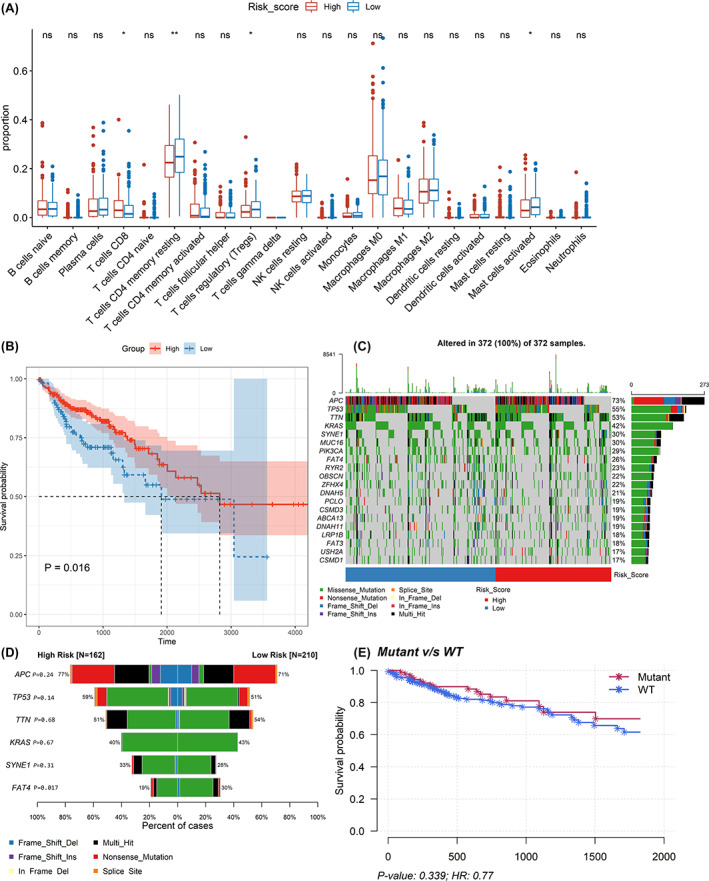
Analyzing the immune cell types and mutation profiles in high‐ and low‐risk groups based on the whole dataset. (A) Comparing the difference of the proportions of immune cells between low‐risk group and high‐risk group using Wilcoxon test. The values of *p* were labeled above each boxplot with asterisks. (*p < .05, **p < .01). (B) The Kaplan–Meier analysis of the relationship between the level of T cells CD4 memory resting with patients' OS. (C) The mutation profiles of colon cancer patients in high‐ and low‐risk groups. (D) Comparison of the mutation rate between high‐risk group and low‐risk group. (E) The association between the *FAT4* status and patients' OS.

**FIGURE 6:**
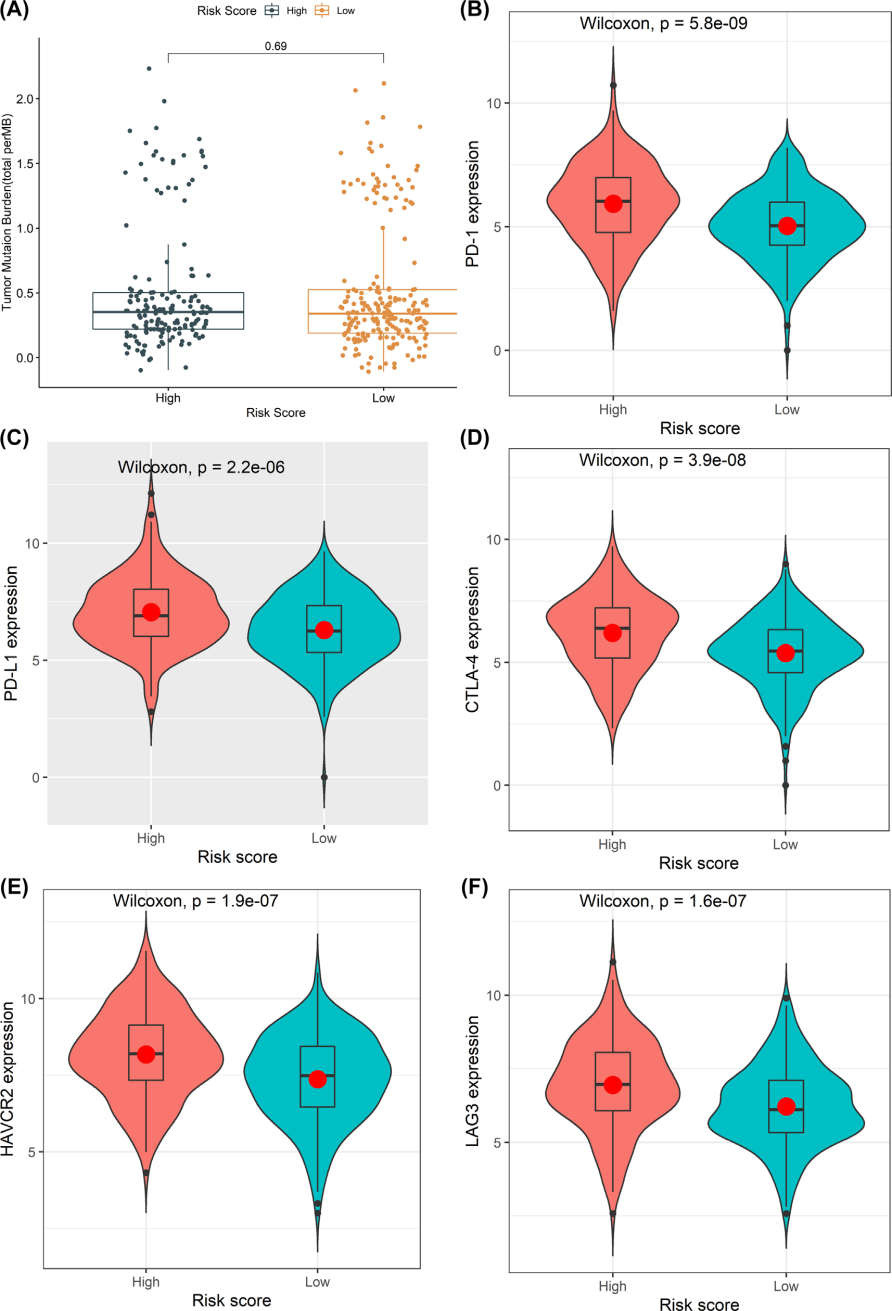
Exploring the tumor immune microenvironment in patients with colon cancer. (A) The difference of TMB between the high‐risk group and the low‐risk group. (B–F) Comparison of the expression levels of the immune checkpoints and their ligands between the high‐risk score group and low‐risk score group. (B) the expression of *PD‐1*, (C)The expression of *PD‐L1*, (D) the expression of *CTLA‐4*, (E) the expression of *HAVCR2*, (F) and the expression of *LAG3*.

Degrees of presence for 22 immune cell populations within individual CC cases were estimated using CIBERSORT algorithm, before statistically comparing low‐risk and high‐risk groups concerning their immune cell proportions. Based on the results, it was noted that significant differences occurred in activated mast cells, regulatory T‐cells (Tregs), CD4 and CD8 T‐cells (*p* < .05, Figure 5A). Among them, poor OS was associated with upregulated CD4 T‐cell presence (*p* = .016, Figure 5B). Mutation profiling for individual CC cases were also evaluated, with top 20 significantly mutated genes being *USH2A, FAT3, LRP1B, DNAH11, ABCA13, CSMD3, PCLO, DNAH5, ZFHX4, OBSCN, RYR2, FAT4, PIK3CA, MUC16, SYNE1, KRAS, TTN, TP53, APC*, and *CSMD1* (Figure 5C). High‐risk cohort exhibited significantly low mutation rates for *FAT4* compared with the low‐risk one (*p* = .017, Figure 5D). Notwithstanding, no major links existed across *FAT4* expression and patients' OS (*p* = .339, Figure 5E). Furthermore, TMB for each patient was calculated but did not exhibit major variations across groups (*p* = .69, Figure 6A).

Moreover, both groups were compared with the *Wilcoxon* test to determine their expression levels for immune checkpoints together with linked ligands. In this case, dataset outcomes demonstrated that high‐risk scoring cases had upregulated *PD‐L1* (*P* = 5.8e^−9^), *PD‐1*(*P* = 2.2e^−6^), *CTLA‐4* (*P* = 3.9e^−8^), *HAVCR2* (*P* = 1.9e^−7^), and LAG3 (*P* = 1.6e^−7^) (Figure 6B–F).

### 3.5 The drug sensitivity in high‐risk and low‐risk groups depending upon generated prognostic model

**FIGURE 7:**
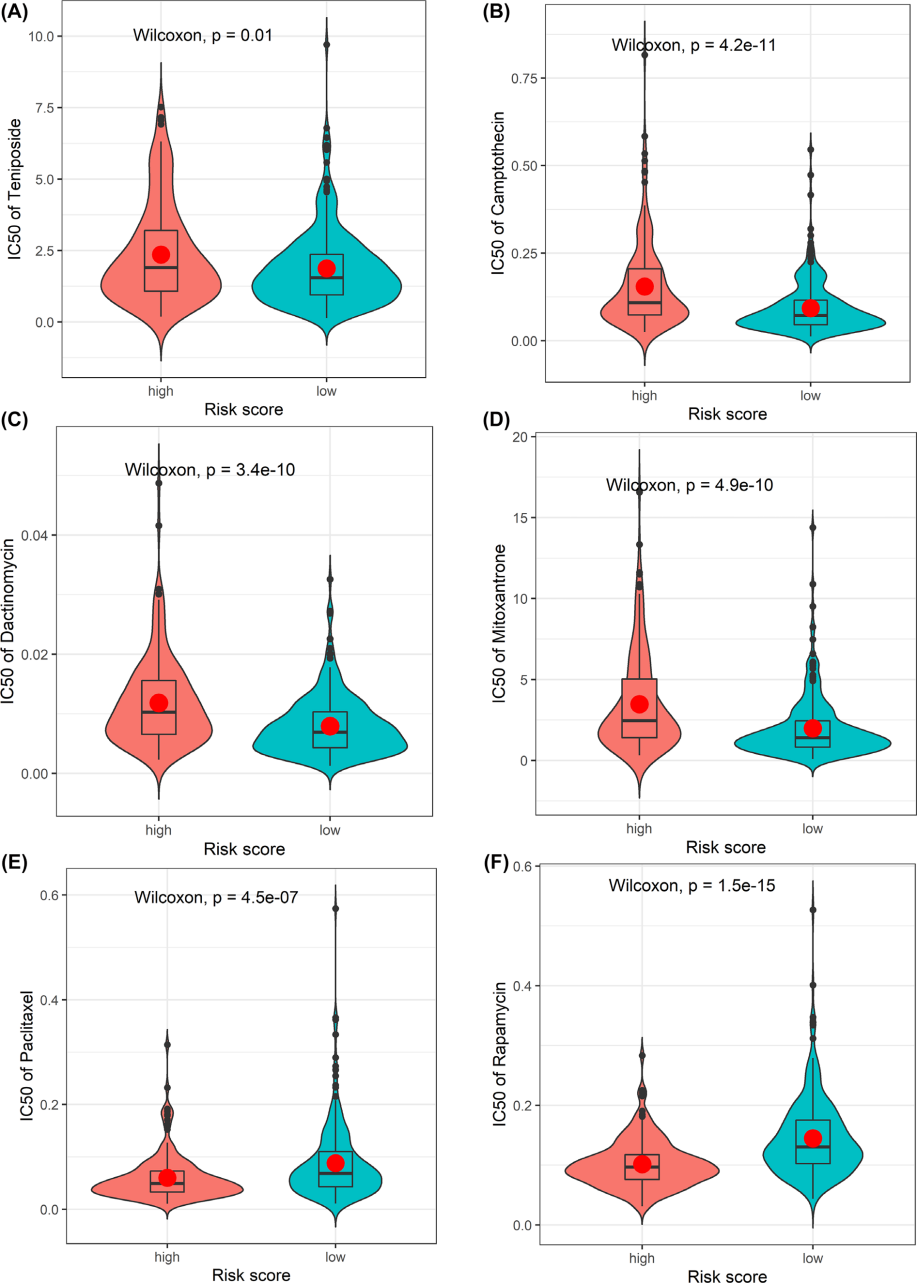
The differences of estimated IC50 of 6 representative drugs between high‐risk and low‐risk groups. (A) the IC50 of Teniposide, (B) the IC50 of Camptothecin, (C) the IC50 of Dactinomycin, (D) the IC50 of Mitoxantrone, (E) the IC50 of Paclitaxel, (F) the IC50 of Rapamycin.

IC50 levels for 198 chemotherapy drugs or inhibitors were predicted in all cases within the TCGA‐COAD cohort, and of these, 43 drugs were found to be sensitive (Table S3). In addition, the two groups of patients were compared, with six representative drugs found to exhibit significant differences (Figure 7A–F). It was found that Dactinomycin, Mitoxantrone, Teniposide, and Camptothecin may be candidate drugs for treating cases within low‐risk cohort, while Paclitaxel and Rapamycin could be suitable in cases within high‐risk cohort.

## 4 DISCUSSION AND CONCLUSION

Previous literature summarized m6A methylation to be implicated within multiple human cancers.^19^ At the same time, lncRNAs may not only serve as potential signatures of prognosis but may also be implicated within carcinogenesis and progression of multiple tumors.^20^ However, limited studies have explored functions for m6A‐related lncRNAs within prognoses and CC immune microenvironment. Therefore, an m6A‐related lncRNAs prognosis‐related model and its effects upon immune microenvironments in CC is required.

Using univariate or multivariate Cox regression, LASSO Cox as well as Pearson correlation analyses, 21 m6A‐related lncRNAs were identified, of which 11 were selected as potential prognostic signatures (*AL139383.1, WDR86‐AS1, AC007743.1, LCMT1‐AS1, AL135960.1, AP003068.4, AC004832.5, AL513123.1, MIR4713HG, AL512306.3, AC104564.4,* and *AC007128.1*). Among them, half of the prognostic lncRNAs were significantly down‐regulated in MSI‐H group (*p* < .05, Figure 3J), which may indicate that the high instability of microsatellite status inhibits the expression of lncRNAs. In particular, unlike 10 of these which were found to be risk factors (HR < 1) (i.e., higher expression leads to worse OS), AL139383.1 was protective (HR > 1). An intronic variant of *AL139383.1* was also show its link to OS in CC cases,^21^ while a previous study reported that *AL513123.1* was overexpressed and, as such, was of potential prognostic value in breast cancer.^22^ Similarly, *MIR4713HG* was experimentally shown to be significantly upregulated in oral tongue squamous cell carcinoma (OTSCC) tissues, thereby making it a potential indicator of prognosis for OTSCC patients, although *MIR4713HG* could act as a risk factor that accelerates cell proliferation and metastasis.^23^ Previous studies suggested that *AC007128.1* could also be considered of prognostic value for various cancer patients, including those with esophageal cancer,^24^ esophageal squamous cell carcinoma,^25^ and bladder cancer.^26^ However, research is still lacking regarding the link between these lncRNAs and the prognosis of colon cancer, and as such, lncRNAs may represent novel prognostic indicators for patients with colon cancer.

This work was not without limitations. First, in validating the m6A‐related prognostic model, only dataset from the TCGA‐COAD cohort was used as additional external datasets of the target lncRNAs from other public databases could not be acquired for validating the robustness of the model further. Furthermore, the molecular functions of these lncRNAs as well as the mechanism through which they influence colon cancer's prognosis need to be further investigated.

Overall, a robust m6A‐related prognostic model was established to explore TIM characteristics and drug resistance potential. These findings could help for prognosis prediction in CC cases, including the development of potential treatments.

## AUTHOR CONTRIBUTIONS

**Lichao Cao:** Conceptualization (equal); data curation (lead); formal analysis (lead); methodology (lead); resources (lead); validation (lead); writing – original draft (lead); writing – review and editing (lead). **Shenrui Zhang:** Data curation (supporting); investigation (supporting); resources (equal); writing – review and editing (supporting). **Ying Ba:** Conceptualization (supporting); data curation (supporting); investigation (supporting); writing – review and editing (supporting). **Hezi Zhang:** Conceptualization (equal); data curation (supporting); methodology (equal); supervision (supporting); writing – review and editing (supporting).

## CONFLICT OF INTEREST STATEMENT

Authors Hezi Zhang, Ying Ba, and Shenrui Zhang were employed by Shenzhen Nucleus Gene Technology Co., Ltd. Lichao Cao declared that the research was conducted in the absence of any commercial or financial relationships that could be construed as a potential conflict of interest.

## ETHICS STATEMENT

Not applicable.

## Supporting information


**TABLE S1.** Detailed information of DELs.

**TABLE S2.** Detailed information of the correlation between DELs and m6A‐related genes.

**TABLE S3.** The IC50 of the 43 relatively sensitive drugs for patients with colon cancer.

## Data Availability

The data that support the findings of this study are openly available in the UCSC Xena (https://xenabrowser.net/datapages/).
